# A Bibliometric Analysis Based on Web of Science: Current Perspectives and Potential Trends of SMAD7 in Oncology

**DOI:** 10.3389/fcell.2021.712732

**Published:** 2022-02-18

**Authors:** Xueying Huang, Zhiying Yang, Jinning Zhang, Ruojiao Wang, Jiahui Fan, Heng Zhang, Rong Xu, Xia Li, Siying Yu, Linna Long, He Huang

**Affiliations:** ^1^ Department of Histology and Embryology, Xiangya School of Medicine, Central South University, Changsha, China; ^2^ Changsha Health Vocational College, Changsha, China; ^3^ Department of Histology and Embryology, School of Basic Medical Science, Xinjiang Medical University, Urumqi, China

**Keywords:** bibliometrics, visualization, SMAD7, oncology, citespace, VOSviewer, web of science, citation

## Abstract

**Background:** The number of publications on SMAD7 in the field of oncology is increasing rapidly with an upward tendency. In most cases, the mechanisms of carcinogenesis usually relate to disorders of signaling activity. Considering the crucial role of SMAD7 in the crosstalk of multiple signaling pathways, it is necessary to clarify and define the dominant research topics, core authors, and their cumulative research contributions, as well as the cooperative relationships among documents or researchers.

**Methods:** Altogether, 3477 documents were retrieved from the Web of Science Core Collection with the following criteria: TS= (SMAD7 OR SMAD7-protein OR Small-Mothers-Against-Decapentaplegic-7) refined by WEB OF SCIENCE CATEGORY (ONCOLOGY) AND [excluding] PUBLICATION YEARS (2021) AND DOCUMENT TYPES (ARTICLE OR REVIEW) AND LANGUAGES (ENGLISH) AND WEB OF SCIENCE INDEX (Web of Science Core Collection, SCI), and the timespan of 2011–2020. Bibliometric visualization analysis was conducted with CiteSpace and VOSviewer.

**Results:** The number of documents grew each year. A total of 2703 articles and 774 reviews were identified from 86 countries/regions, 3524 organizations, 928 journals, and 19,745 authors. China was the most prolific country, with 1881 documents. Contributions from China, the United States, and Germany were the most substantial. The most influential author was Lan Huiyao at The Chinese University of Hong Kong, with 24 publications and 2348 total citations. The bibliometric analysis showed that multilateral cooperation among diverse institutions or investigators was beneficial to high-quality outputs. The keyword “PPAR-gamma” exhibited the strongest burst in recent years, suggesting a potent research focus in the future.

**Conclusion:** Research on SMAD7 in oncology is continuously developing. Bibliometrics is an interesting tool to present the characteristics of publication years, main authors, and productive organizations in a visualized way. It is worth mentioning that a prospective focus might be the specific mechanism of the interaction of PPAR-gamma with SMAD7 in oncology. In all, bibliometric analysis provides an overview and identifies potential research trends for further studies in this academic field.

## Introduction

Small mothers against decapentaplegic (SMAD) protein family members are commonly divided into three main categories according to their specific mechanisms and participation in diverse signaling pathways. First, Smad1, Smad5, and Smad8 (bone morphogenetic protein, BMP pathway), as well as Smad2 and Smad3 (transforming growth factor, TGF pathway) are classified as receptor-regulated SMADs (R-SMADs). SMAD4 is a common-partner SMAD (Co-SMAD). The inhibitory SMADs (I-SMADs) include SMAD6 and SMAD7 (Derynck et al., 1998).

Cytokines of the TGF signaling pathway and BMP signaling pathway are essential elements for normal development and physiology, with roles in regulating cell proliferation, differentiation, apoptosis, and migration (Heine et al., 1987; Kishigami and Mishina, 2005). As critical mediators of the TGF-beta and BMP pathways, SMAD proteins not only can directly regulate the transcription of specific genes in these signaling pathways (Derynck et al., 1998) but also participate in crosstalk with other signaling pathways. Perturbation of signaling pathways induces disease pathogenesis, such as wound healing (Sporn et al., 1983), fibrosis (Meng et al., 2016), and cancer (Dituri et al., 2019).

SMAD7 is an inhibitory SMAD family protein. It can negatively regulate signal transduction pathways such as the TGF-beta and BMP pathways by inhibiting the phosphorylation of receptor-regulated SMAD proteins (Itoh and Ten, 2007). Aside from its functions in normal physiological regulation, unbalanced relationships between SMAD7 and signaling pathways exist in pathologic conditions (Dituri et al., 2019; Ma et al., 2018; Meng et al., 2016). This is especially relevant because many mysteries related to carcinoma remain unsolved. Bibliometric studies on published results will help to summarize and evaluate current research achievements and, moreover, serve as an effective reference for further research in the field of oncology.

Bibliometric analysis is widely used to calculate the scholarly impact of any scientific productions in a visualization way (PRICE, D. J. d. S., 1965). It includes obtaining, processing, and managing quantitative data from existing publications. Despite its methodological limitations, bibliometric analysis is a useful tool for assessing the scientific relevance of a given field and is used in an extensive range of universities to assess research performance.

However, we find that few bibliometric studies have been conducted on SMAD7. Although some publications have made numerous important conclusions related to SMAD7 in oncology, there has been no attempt to map out the entire body of SMAD7 research in a systematic manner. After searching for “SMAD7 OR SMAD7-protein OR Small-Mothers-Against-Decapentaplegic-7” and “Bibliometrics OR Bibliometric” in the Web of Science Core Collection, no results were retrieved. Although it is possible some bibliometric publications about SMAD7 did not include “bibliometric(s)” in their titles or abstracts, it can be said that few bibliometric studies have been conducted on SMAD7.

Based on the hypothesis that SMAD7 deregulation is linked to several types of cancers (Ha et al., 2019; Tong et al., 2020; Troncone and Monteleone, 2019), we aimed to perform a bibliometric analysis on SMAD7 research in the scope of oncology. With the help of visualization analysis software such as CiteSpace and VOSviewer, the significance, centrality, and quality of publications, as well as their correlations, can be intuitively observed in the relationship network (Synnestvedt et al., 2005; van Eck and Waltman, 2010). To an extent, mathematical and statistical methods are useful to analyze the progress of a specific academic field and to predict potential development trends in the scientific community (Chen, 2006; van Eck and Waltman, 2010).

In this study, we retrieved research literature on SMAD7 in the field of oncology from the Web of Science Core Collection and then utilized CiteSpace and VOSviewer for data analysis. Our study aimed to provide an overview of these publications, to summarize and clarify the mainstream research topics, and more importantly, to highlight emerging topics to help researchers incorporate new ideas in their future studies.

## Materials and Methods

### Data Strategy and Selection Criteria

The Web of Science Core Collection originated in 1985. It contains world-class indexes such as Science Citation Index Expanded (SCIE), Social Sciences Citation Index (SSCI), Arts and Humanities Citation Index (A&HCI), etc. Two of the major strengths of the Web of Science Core Collection are reference tracing and citation reporting. Aside from enabling search within leading academic journals, books, and citation networks, it also has powerful capabilities for tracing reference and citation activity to identify outputs and trends in a given research area.

“SMAD7 protein” was used as the search term in the Medical Subject Headings (MeSH) database (https://www.ncbi.nlm.nih.gov/mesh). To research target documents as comprehensively as possible, terms similar to “SMAD7”, such as “SMAD7 protein” and “Small mothers against decapentaplegic 7,” were also all searched in the Web of Science Core Collection.

**FIGURE 1:**
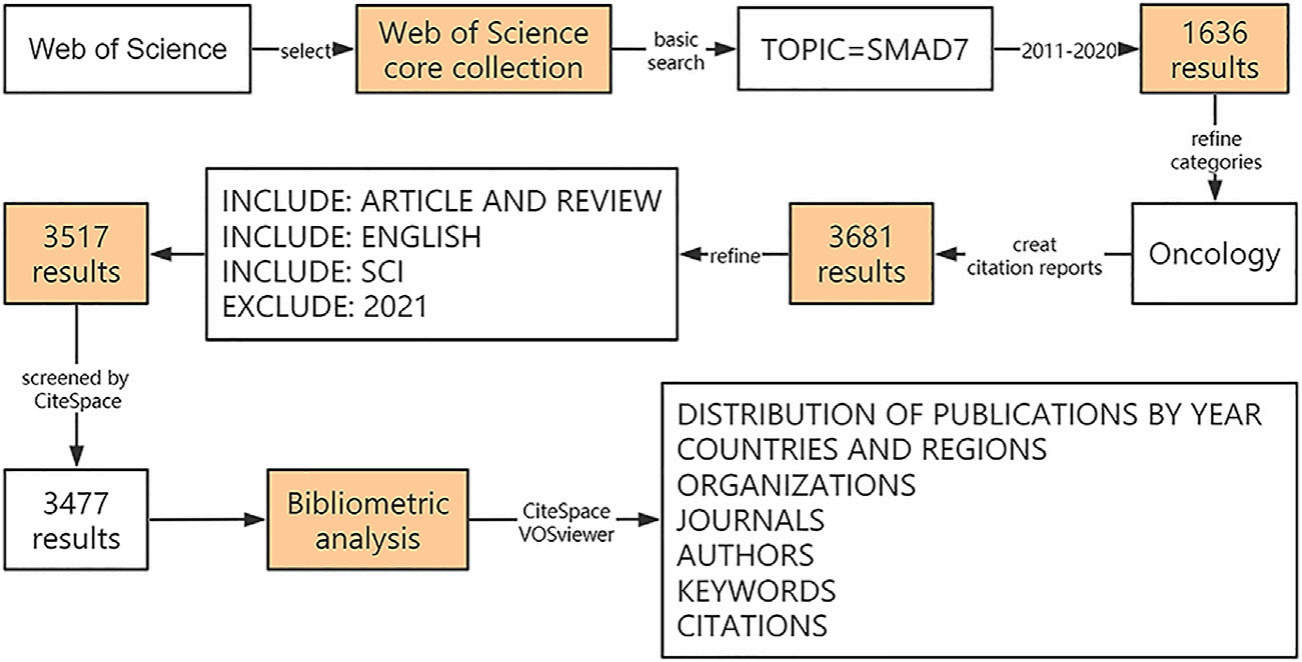
Detailed flowchart steps of the search strategy in the screening of publications.

Then, the search strategy used is shown in Figure 1: TS= (SMAD7 OR SMAD7-protein OR Small-Mothers-Against-Decapentaplegic-7) refined by WEB OF SCIENCE CATEGORY (ONCOLOGY) AND [excluding] PUBLICATION YEARS (2021) AND DOCUMENT TYPES (ARTICLE OR REVIEW) AND LANGUAGES (ENGLISH) AND WEB OF SCIENCE INDEX (Web of Science Core Collection. SCI), and the timespan of 2011–2020. All of keywords mentioned above were not used with “*” to truncate.

Overall, a total of 3517 documents were collected from the Web of Science Core Collection according to the above criteria. After using CiteSpace to remove duplicates or publications with unclear year of publication, the remaining 3477 documents were subjected to visualization analysis. This search was completed on February 3, 2021.

### Methodology

Retrieval results regarding SMAD7 in the field of oncology were analyzed with the distribution of publication years, countries and regions, organizations, journals, core authors, keywords, and key references. Bibliometrics on SMAD7 were visualized by CiteSpace (Version 5.7 R2; https://citespace.podia.com), VOSviewer (Version 1.6.16; https://www.vosviewer.com), the Bibliometrics Online Analysis Platform (https://bibliometric.com), and Microsoft Excel 2016.

Price’s Law is the most widely used index to access the growth situation of current outcomes in a specific country or a given academic discipline (PRICE, D. J. d. S., 1965). Based on the formulas and correlation indexes from the number of publications by year distribution, papers’ increase pattern will be revealed as the exponential or linear style. In Price’s Law, most scientific growth models are supposed to show an exponential development.

CiteSpace is software that can be used to analyze co-occurrence and the centrality of cooperation networks of countries/authors/institutions (Chen, 2006). In this study, LLR was chosen as the CiteSpace analysis method. The circumference of each node presents its number of documents, and the proportion of the outermost ring means its centrality. Centrality generally shows the importance of a node in a network. Sigma is the index based on both centrality and burst to indicate the key role in citation activity. The thickness of the line between two nodes indicates the strength of the association. VOSviewer can be used to visualize scientific landscapes by network/overlay/density patterns with the Linlog/modularity method (van Eck and Waltman, 2010). For each node, the weight is assessed by citations or documents, and the color indicates the average published year or cluster type depending on the selected analysis pattern. The Bibliometrics Online Analysis Platform is an online platform that can be used to perform a cooperation network among countries or regions. Microsoft Excel 2016 was the basic tool used to import and sort data, as well as for tabulation.

## Results

### Distribution of Publications by Year

**FIGURE 2:**
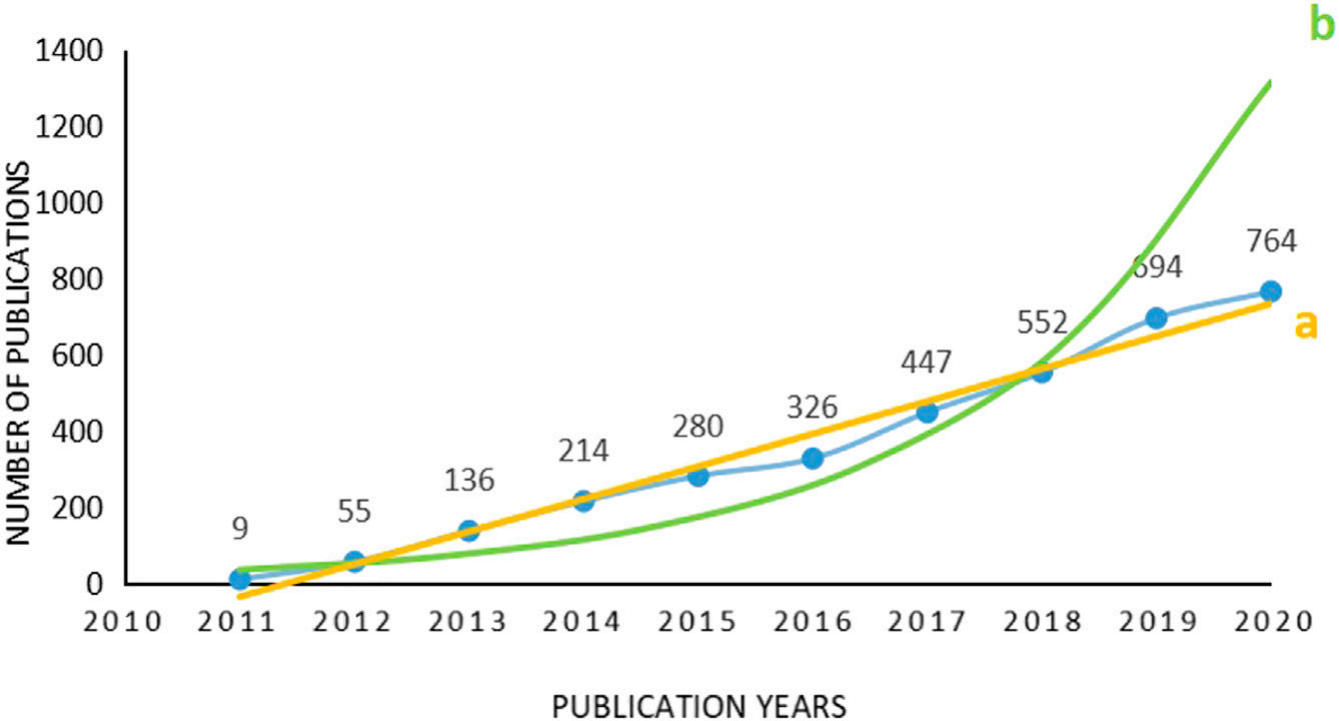
Distribution of publications on SMAD7 in the field of oncology according to the year. Footnote: The number of publications increased gradually year by year from 2011 to 2020, and the number of publications rose relatively sharply from 2016 to 2019, then reached a peak in 2020. Linear adjustment (a): y = 85.412x-171800, *r* ^2^ = 0.9819. Exponential adjustment (b): y = 22.512e^0.4066x^, *r* ^2^ = 0.8125.

There were 2703 articles (78.89%) and 774 reviews (22.26%) among the 3477 included documents, averaging 348 publications per year. The distribution of publication number by year, presented in Figure 2, shows an increasing growth trend. Only nine publications about SMAD7 were published in 2011, and the number of publications showed a nearly sixfold increase, to 55, in the next year. The 4 years from 2016 to 2019 witnessed a relatively sharp rise in the number of publications. Then, the document number reached a peak in 2020. To ensure whether the growth of publications about SMAD7 in oncology conforms to Price’s Law, exponential and linear adjustments were performed with the acquired data. The equation y = 22.512e^0.4066x^ (with a correlation coefficient of 0.8125) was obtained from its exponential curve, and another equation y = 85.412x-1719800 (with a correlation coefficient of 0.9819) followed by a linear fit. In summary, the continuous increase in publications over time indicates that SMAD7 is receiving significant attention in the field of oncology.

### Countries and Regions

**FIGURE 3:**
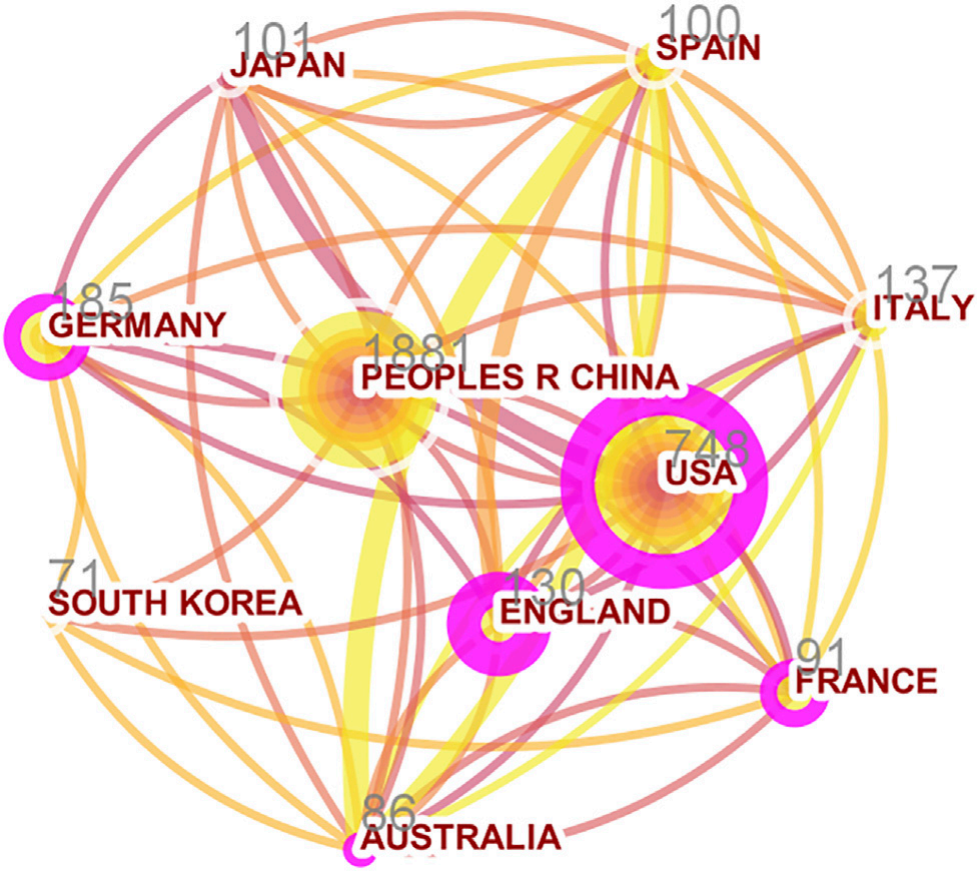
Analysis of cooperation among the top 10 countries/regions with the highest documents. Footnote: The circumference of each node presents its number of documents, and the proportion of the outermost ring means its centrality.

This research involved a set of 86 countries or regions. The top 10 most productive countries or regions are presented in Table 1. The major evaluation indices include the number of documents, number of citations, total link strength, links, and centrality. China was the country with the largest number of publications (n = 1881, 54.10%), followed by the United States (n = 748, 21.51%) and Germany (n = 185, 5.32%). As shown, the total number of documents published in China represented over 50% of all documents. Cooperative relationships among different countries/regions are shown in Figure 3. The purple circles around nodes represent centrality in the network, which indicates a pivotal role in the cooperative relationships among countries. Countries with centrality values over 0.1 include the United States, England, Germany, Australia, and France. Although the number of publications in China was the largest, the total citations and centrality of China were far lower than those of the United States. The abovementioned results indicate that the United States still dominates SMAD7 research and participates in more exchange and cooperation in the field of oncology.

**TABLE 1:** More details about the most productive 10 countries/regions with documents on SMAD7 in the field of oncology.

NO.	Country	Documents	Sigma	Centrality
1	China	1881	1	0.01
2	United States	748	10.38	0.67
3	Germany	185	1	0.15
4	Italy	137	1	0
5	England	130	1	0.31
6	Japan	101	1	0
7	Spain	100	1	0.34
8	France	91	1.47	0.12
9	Australia	86	2.03	0.33
10	South Korea	71	1	0.01

**FIGURE 4:**
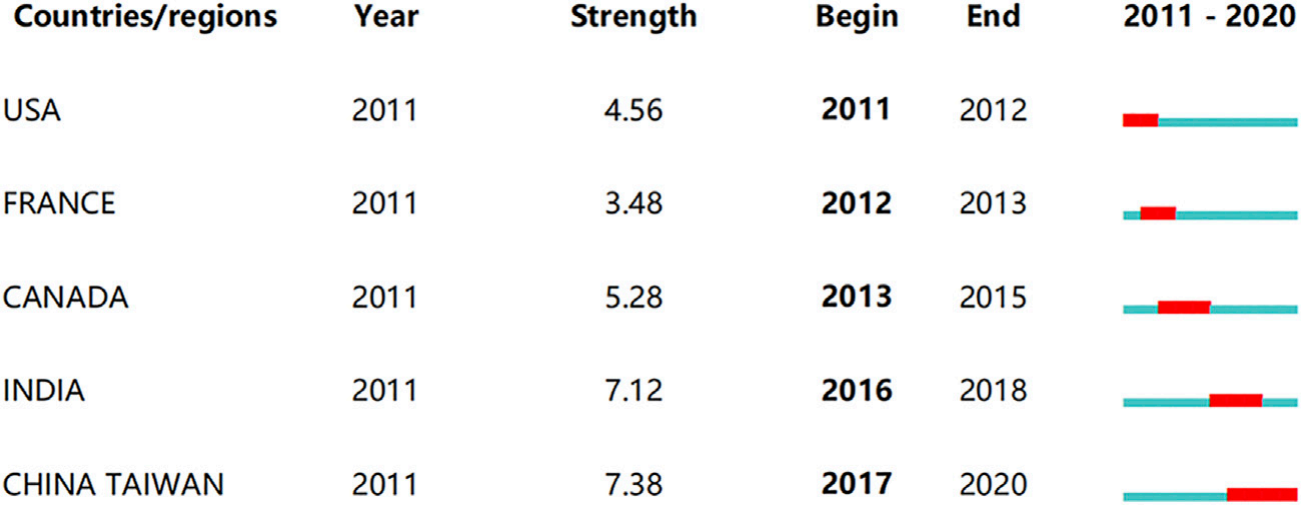
Top five countries/regions with the strongest citation bursts. Footnote: The strongest citation burst means that a variable changes greatly in a short period. Red bars indicate the duration of the burst.

The top five countries or regions with the strongest citation bursts are shown in Figure 4. Taiwan had the largest burst length, 7.38, which started from 2017 to 2020, indicating that studies on SMAD7 in the field of oncology emerged in Taiwan from 2017 to 2020. France had the shortest burst length, 3.48, which rose beginning in 2012 and ended in the next year, suggesting that there was a short burst of research on SMAD7 in France for 1 year.

### Organizations

According to VOSviewer analysis, 3524 different organizations were involved in the research worldwide. After eliminating disjointed organizations from the 339 organizations that met the threshold, 334 organizations remained. The top 10 prolific organizations ranked by document numbers are shown in Table 2 with total citations and total link strength. As shown, all top 10 institutions are in China. Nanjing Medical University was the most prolific organization, with 94 publications, accounting for 2.67%, followed by Sun Yat-Sen University (n = 90, 2.55%) and Shanghai Jiao Tong University (n = 83, 2.36%).

**TABLE 2:** Institutions ranked by literature quantity.

NO.	Institution	Links	Total link strength	Documents	Total citations
A. Information of the top 10 most productive institutions					
1	Nanjing Medical University	44	72	94	1056
2	Sun Yat-sen University	54	120	90	1220
3	Shanghai Jiao Tong University	43	76	83	963
4	Zhejiang University	45	76	79	931
5	Harbin Medical University	30	77	73	1132
6	Huazhong University of Science and Technology	31	66	72	981
7	Fudan University	35	52	66	715
9	Zhengzhou University	20	60	64	803
8	China Medical University	27	44	64	512
10	Xi`an Jiaotong University	29	41	62	873
B. The institutions with total citations over 2000					
:	:	:	:	:	:
14	The Chinese University of Hong Kong	43	86	51	2803
:	:	:	:	:	:
23	Anhui Medical University	18	32	39	2114
:	:	:	:	:	:
28	German Cancer Research Center	61	266	35	2313
:	:	:	:	:	:
56	The University of Southern California	51	143	22	4215
:	:	:	:	:	:
81	The University of California at San Francisco	26	35	16	5348

Among the top 10 organizations, three universities produced more than 1000 citations. The order from highest to lowest was Sun Yat-Sen University (citations = 1220), Harbin Medical University (citations = 1132), and Nanjing Medical University (citations = 1056). Among them, Sun Yat-Sen University had both the largest total link strength and the largest total number of citations, 120 and 1220, respectively. Significantly, although Harbin Medical University did not produce as many documents as the top three institutions, its citations numbered over 1000, ranking second, which means this institution produced considerable research with a high number of citations. In addition, the most common publication year for the top 10 institutions was 2017 (n = 8, 80%), demonstrating that these institutions have remained active in recent years.

The organizations with over 2000 citations, excluding the top 10, are listed in Table 2. This list includes The Chinese University of Hong Kong (citations = 2803, n = 51), Anhui Medical University (citations = 2114, n = 39), German Cancer Research Center (citations = 2313, n = 35), etc. It is worth noting that the University of California at San Francisco had the largest number of citations among all 334 organizations, reaching 5348 citations with 16 documents. Similarly, The University of Southern California published 22 documents with a total of 4215 citations. Both organizations with the highest total citations are located in the United States.

### Journals

The publications related to SMAD7 in the field of oncology were published in 928 journals from 2011 to 2020. Through analysis of the journals involved, it is possible to identify the main journals in a particular academic field. The top 10 journals with the largest number of publications and their 2019 impact factors are presented in Table 3. Impact factor (IF) is a quantitative measure utilized to evaluate the significance of absolute or total citation frequencies (https://clarivate.com/webofsciencegroup/essays/impact-factor/). As shown, the 2019 impact factors of these 10 journals range from 1.785 to 6.126, averaging 3.704. The 2019 impact factor of *Cancers* was the highest and that of *Experimental and Therapeutic Medicine* was the lowest. The top three journals with the largest number of documents were *PLoS One*, *Oncotarget,* and *International Journal of Molecular Sciences*, so substantial numbers of studies may be found in these journals. Regarding the reference value of annual impact factors, *Cancers*, *International Journal of Molecular Sciences,* and *Biomedicine & Pharmacotherapy* may be considered the authoritative journals in this field. Judged from both document number and impact factors, the *International Journal of Molecular Sciences* may be the most influential journal. More detailed, to check out the inclusiveness and acceptance of publications focused on SMAD7 in each journal, the ratios were conducted by “number of papers about SMAD7 in oncology/total number of papers,” ranging from 0.000 to 0.143. *PLoS One* was the most productive journal of both total papers (number = 105) and SMAD7 papers (ratio = 15/105).

**TABLE 3:** Top 10 journals with the largest number of documents.

NO.	Journal	2019 IF	Documents	Citations	Ratios*
1	PLoS One	2.740	105	2064	0.143
2	Oncotarget	3.337	99	1878	0.000
3	International Journal of Molecular Sciences	4.556	75	1246	0.120
4	Scientific Reports	3.998	70	922	0.071
5	Molecular Medicine Reports	2.100	59	391	0.000
6	Oncology Letters	2.311	58	284	0.000
7	Biomedicine & Pharmacotherapy	4.545	48	575	0.042
8	Cancers	6.126	46	286	0.000
9	Journal of Cellular Physiology	5.546	38	794	0.136
10	Experimental and Therapeutic Medicine	1.785	37	255	0.135

Ratios*: the number of papers about SMAD7 in oncology/total number of papers in each journal.

### Authors

**FIGURE 5:**
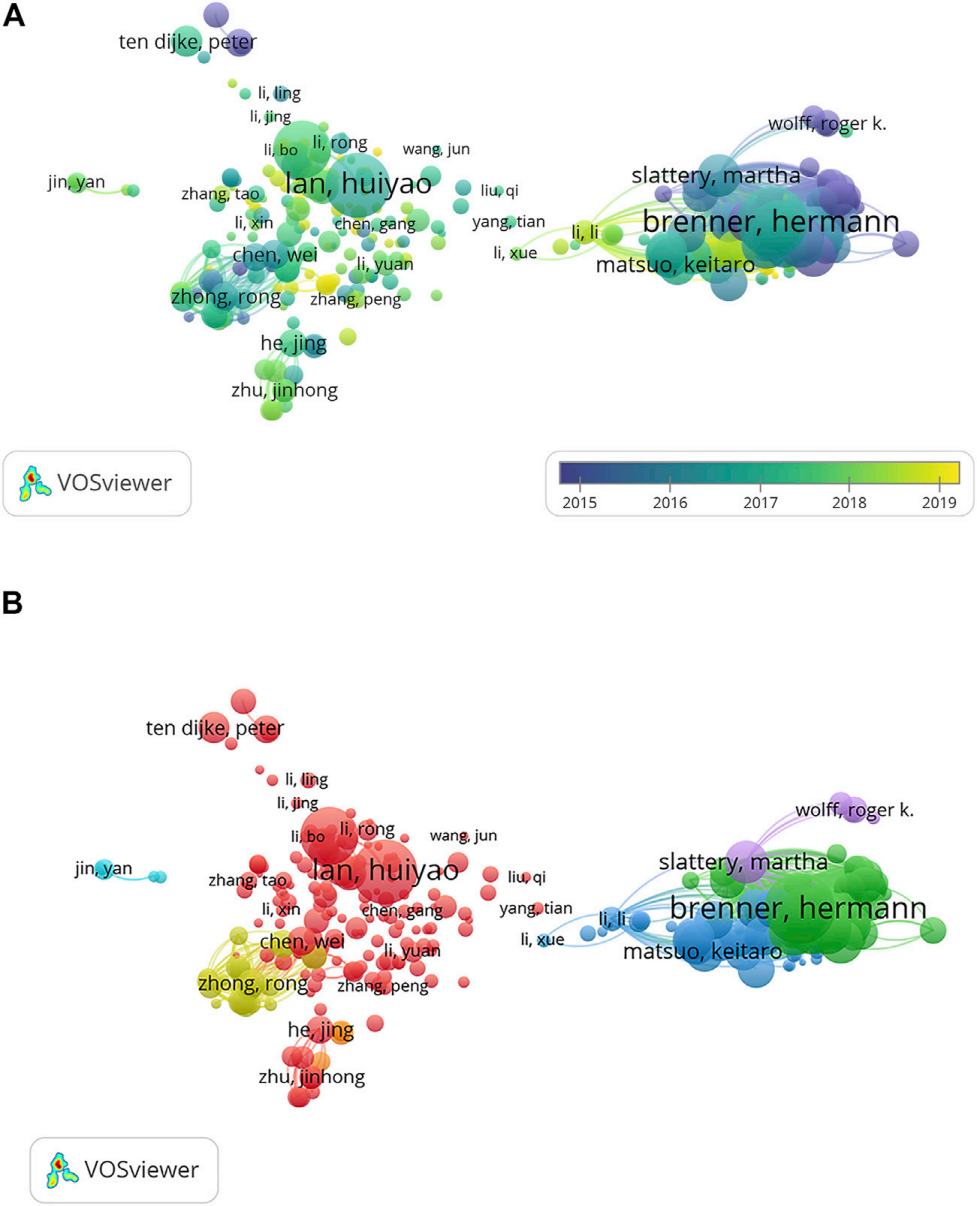
Visualization graphs of co-authorship analysis of authors. **(A)** Overlay visualization of authors involved in studies on SMAD7 in oncology. **(B)** Network visualization of authors involved in studies on SMAD7 in oncology. Figure 5A Footnote: The analysis method was Linlog/modularity in VOSviewer, the weight was citations, and scores were the average publication year. The thickness of the lines indicates the strength of the relationship. The color means the average publication year. Figure 5B Footnote: The total authors were classified into 7 clusters with different colors to show several major cooperative groups.

Altogether, 19,745 authors were involved in producing 3477 related publications. Analysis of authors and co-cited authors contributes to determining core authors and major cooperative relationships in this field. The key authors and their affiliations, number of documents, number of citations, total link strength, and H-index are shown in Table 4. Among these indices, total link strength shows the level of relevance to another researcher, and the H-index provides an estimate of the significance and influence of a certain researcher’s cumulative research contributions (Hirsch, 2005). The authors on this list are generally from prolific countries: China (n = 6), the United States (n = 2), and Germany (n = 2). This analysis also indicates that Lan Huiyao was the top researcher, with a total of 2348 citations. Brenner Hermann had the highest H-index, 122. From the visualization analysis in Figure 5A, we can observe that the top authors (Lan HY, Meng XM, and Li J) in the list had a stable co-occurrence relation. Among the 19,745 authors identified, 355 met the threshold for inclusion. After excluding authors who were unconnected from others, a total of 304 authors remained. Network visualization was performed to show the dominant co-authorship relations of authors, and seven different author clusters are presented in Figure 5B. The first group was mainly dominated by Lan Huiyao, and the second group was dominated by Brenner Hermann. The third author cluster mainly included Chen Wei. In addition, from the overlay visualization graph, we can see that there are still numerous emerging groups researching SMAD7 in the field of oncology.

**TABLE 4:** Pivotal authors of documents of SMAD7 in the field of oncology from 2011 to 2020.

NO.	Author	Affiliation	H-index	Total link strength	Documents	Citations
1	Lan Huiyao	The Chinese University of Hong Kong	82	39	24	2348
2	Meng Xiaoming	Anhui Medical University	34	64	15	1825
3	Hermann Brenner	Germany Cancer Research Center	122	15	5	1525
4	Li Jun	Anhui Medical University	45	66	22	580
5	Patrick M Tang	The Chinese University of Hong Kong	19	25	9	569
6	Dijke P Ten	Leiden University	114	1	11	494
7	Ogino Shuji	Harvard Medical School	88	42	10	492
8	Andrew T Chan	Harvard Medical School	32	51	13	464
9	Zhang Lei	Harbin Medical University	13	32	13	432
10	Chen Wei	Huazhong University of Science and Technology	12	131	25	419

### Keywords

**FIGURE 6:**
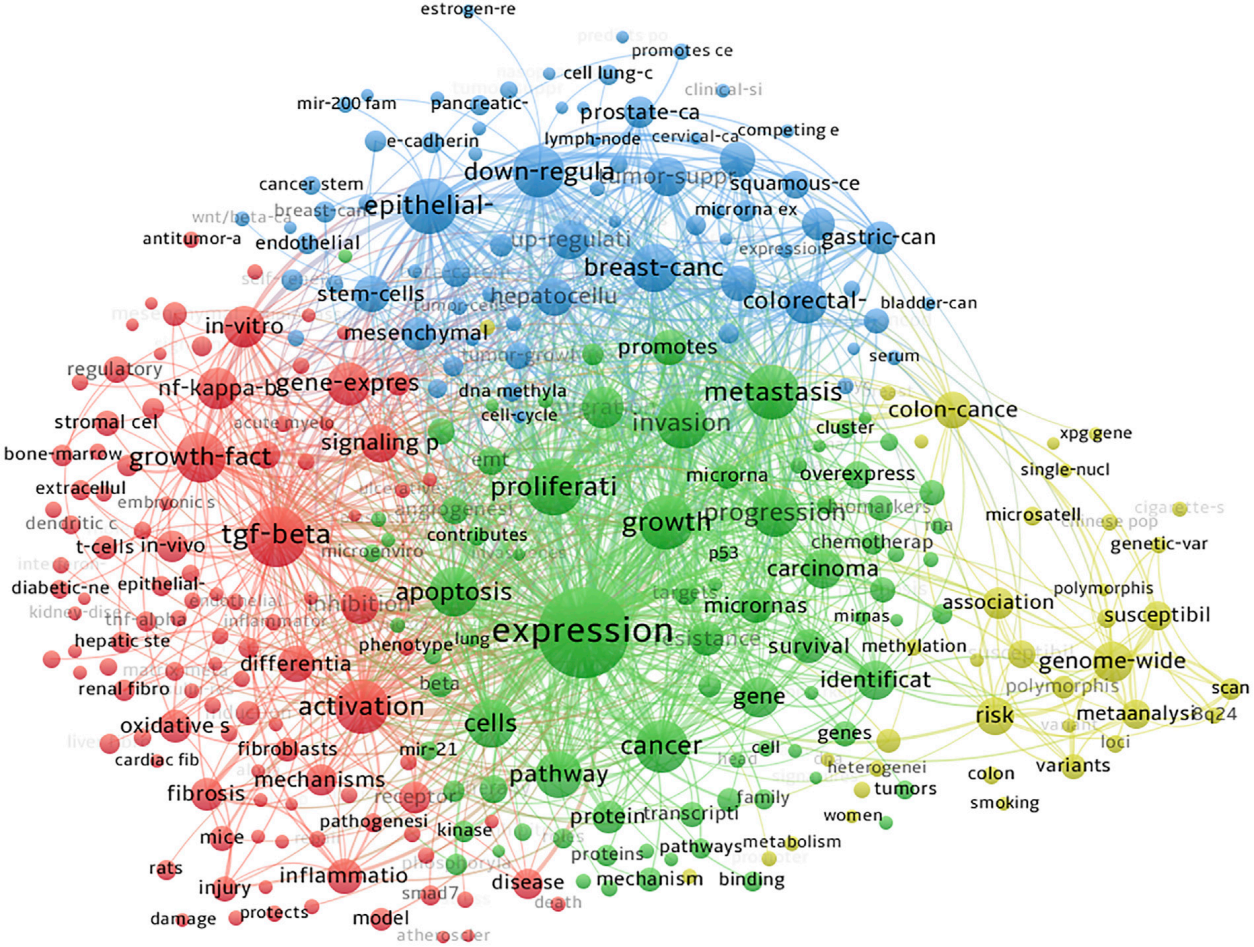
Clustering co-occurrence map of the predominant keywords of studies on SMAD7 in oncology. Footnote: The theme words were classified into 4 clusters, presented by four colors (red, blue, green, and yellow).

A total of 7779 keywords were extracted from titles and abstracts, and 864 items met the threshold. Through analysis of the keywords involved, it is beneficial to clarify the main themes and establish a framework of studies on SMAD7 in an academic field. The top 300 keywords are visualized in Figure 6, showing that theme words were classified into four clusters, represented by four colors (red, blue, green, and yellow). The high-occurrence words include expression, TGF-beta, proliferation, epithelial-mesenchymal transition, and metastasis activation, among others. Most of these keywords denote key molecules, disease patterns, and cell types, as shown in Table 5, which indicate some mainstream topics and frontiers in a particular research area. The top three focused molecules are TGF-beta, NF-kappa-B, and beta-catenin, and the most common diseases involved in studies on SMAD7 include breast cancer, colorectal cancer, and hepatocellular carcinoma. Proliferation, epithelial-mesenchymal transition, and metastasis are the first three physiological or pathological states related to studies on SMAD7.

**TABLE 5:** Top 10 key molecules, states, diseases, and cell types in studies on SMAD7 in oncology.

NO.	Molecule	Occurrence	State	Occurrence	Disease	Occurrence	Cell	Occurrence
1	TGF-beta	413	Proliferation	363	Breast-cancer	245	Stem cells	134
2	NK-kappa-B	179	Epithelial-mesenchymal transition	337	Colorectal cancer	189	Regulatory T-cells	67
3	beta-catenin	68	Metastasis	328	Hepatocellular carcinoma	145	Cancer cells	66
4	IFN-gamma	47	Invasion	281	Colon cancer	137	Stromal cells	58
5	Transforming growth-factor-beta-1	44	Apoptosis	260	Lung cancer	110	Fibroblasts	54
6	Smad7	34	Progression	243	Gastric cancer	109	T-cells	53
7	TNF-alpha	33	Migration	184	Prostate cancer	101	Hepatic stellate cells	41
8	P53	23	Differentiation	131	Squamous cell carcinoma	70	Breast cancer cells	39
9	Necrosis-factor-alpha	22	Inflammation	122	Ovarian cancer	38	Dendritic cells	31
10	PPAR-gamma	22	Fibrosis	107	Pancreatic cancer	35	Endothelial cells	22

Abbreviations: TGF, transforming growth factor; NF, nuclear factor; IFN, interferon; Smad, small mother against decapentaplegic; TNF, tumor necrosis factor; PPAR, peroxisome proliferator activated receptor.

**FIGURE 7:**
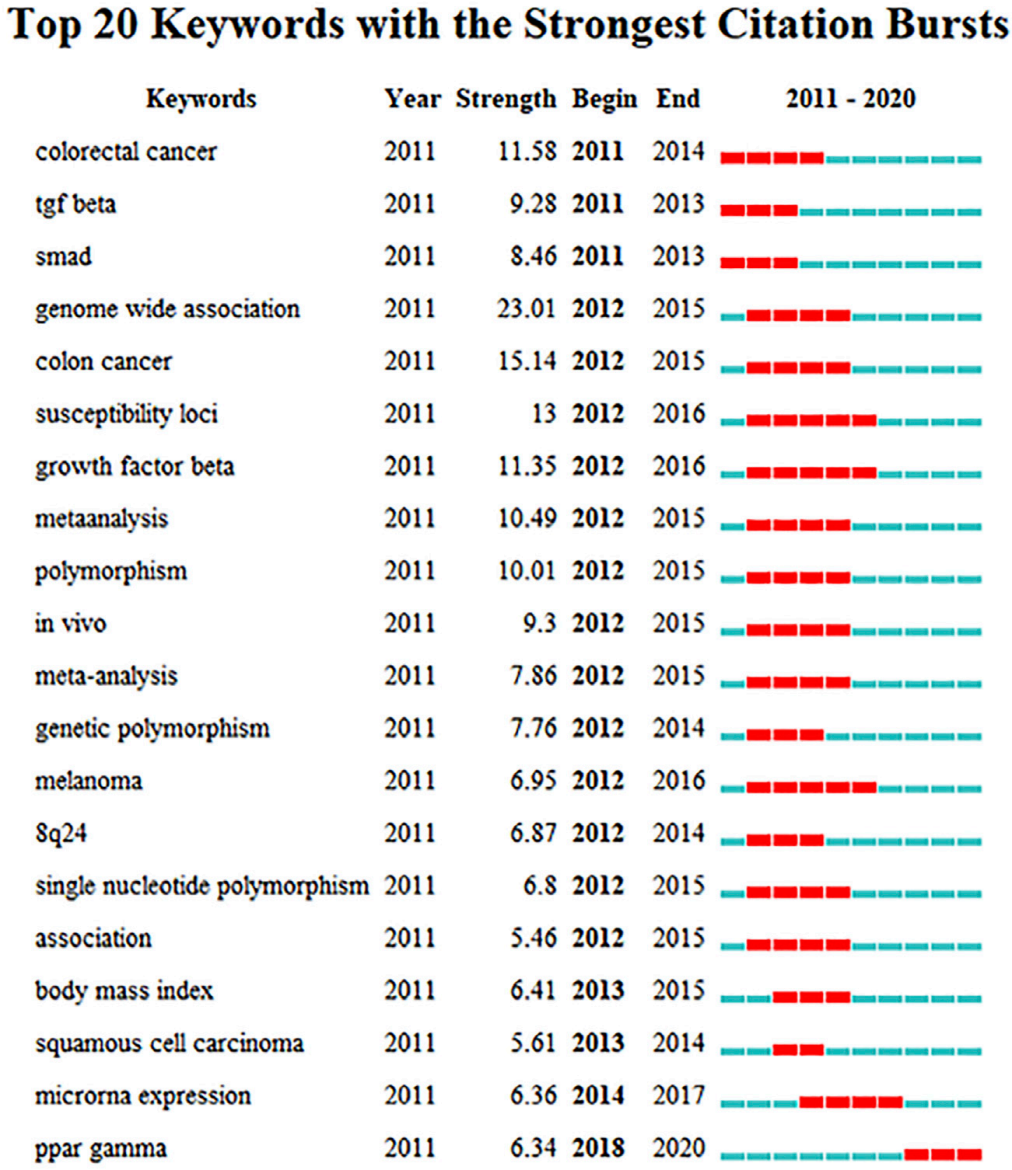
Top 20 keywords with the strongest citation bursts. Footnote: The strongest citation burst means that a variable changes greatly in a short period. Red bars indicate the duration of the burst.

The top 20 keywords with the strongest citation bursts are shown in Figure 7. Genome-wide association had the highest burst length of 23.01, which started in 2012 and ended in 2015. Moreover, items such as susceptibility loci, genetic polymorphism, 8q24, and single-nucleotide polymorphism all suggest trends of research at the genome level. From Figure 7, we can also see that studies on SMAD7 in colorectal cancer, melanoma, and squamous cell carcinoma have risen sequentially in the past decade. Most importantly, PPAR-gamma presented a prominent research trend in the field of oncology from 2018 to the present.

### Citations

The 10 documents with the highest total citation numbers are ranked in Table 6, with a range of 314–3604. It is helpful to sort professional documents in a specific field and to clarify the point in time at which certain items arose in the past. Ranked first, “Molecular mechanisms of epithelial-mesenchymal transition” was published by Samy Lamouille in 2014 and was cited 3604 times in total. On the list, Meng Xiaoming appears twice with “TGF-β: the master regulator of fibrosis” and “TGF-β/Smad signaling in renal fibrosis” in the following year.

**TABLE 6:** Top 10 highly cited documents on SMAD7 in the field of oncology.

NO.	Title	First author	Journal	Total citations	Publication year
1	Molecular mechanisms of epithelial–mesenchymal transition	Samy Lamouille	Nature Reviews Molecular Cell Biology Volume	3604	2014
2	Colorectal cancer	Hermann Brenner	The Lancet	1509	2014
3	TGF-β: the master regulator of fibrosis	Meng Xiaoming	Nature Reviews Nephrology	840	2016
4	Targeting the TGFβ signalling pathway in disease	Rosemary J. Akhurst	Nature Reviews Drug Discovery volume	826	2012
5	Large-scale genotyping identifies 41 new loci associated with breast cancer risk	Kyriaki Michailidou	Nature Genetics	701	2013
6	TGF-β signaling and epithelial–mesenchymal transition in cancer progression	Yoko Katsuno	Current Opinion in Oncology	483	2013
7	The miR-17/92 cluster: a comprehensive update on its genomics, genetics, functions and increasingly important and numerous roles in health and disease	E Mogilyansky	Cell Death and Differentiation	452	2013
8	TGF-β/Smad signaling in renal fibrosis	Meng Xiaoming	Frontiers in Physiology	320	2015
9	Targeting the TGFβ pathway for cancer therapy	Cindy Neuzillet	Pharmacology and Therapeutics	318	2015
10	Regulation of EMT by TGFβ in cancer	Carl-Henrik Heldin	FEBS Letters	314	2012

Apparently, “TGF-beta,” “fibrosis,” and “epithelial-mesenchymal transition” have been mentioned many times in most documents, which indicates that these core molecules and states and their related signaling pathways or mechanisms are critical in research on SMAD7 in oncology.

Additionally, after analysis of the co-citation of cited references, the main references can be retrieved from objective publications. As shown in Table 7, “The miR-106b-25 cluster targets Smad7, activates TGF-β signaling, and induces EMT and tumor initiating cell characteristics downstream of Six1 in human breast cancer” was the top highly cited article with 188 citations. Other cited references included Bartel (2004), Broderick et al. (2007), Tomlinson et al. (2007), Zanke et al. (2007), Massague,(2008), Houlston et al. (2008), Tenesa et al. (2008), Tomlinson et al. (2008), and Houlston et al. (2010). Among the top 10 references, seven articles focused on genome-wide association studies to identify susceptibility loci for colorectal cancer, and the remaining references focused on microRNAs or TGF-beta in cancer. According to the above analysis results, it can be concluded that genome-wide association has consistently been a critical issue in studies on oncology.

**TABLE 7:** Top 10 highly co-citation of cited references on SMAD7 in the field of oncology.

NO.	Title	First author	Journal	Citations	Publication year
1	The miR-106b-25 cluster targets Smad7, activates TGF-beta signaling, and induces EMT and tumor initiating cell characteristics downstream of Six1 in human breast cancer	Anna L. Smith	Oncogene	188	2012
2	MicroRNAs: genomics, biogenesis, mechanism, and function	David P. Bartel	Cell	185	2004
3	TGFbeta in Cancer	Joan Massagué	Cell	117	2008
4	Meta-analysis of genome-wide association data identifies four new susceptibility loci for colorectal cancer	Richard S. Houlston	Nature Genetics	85	2008
5	Genome-wide association scan identifies a colorectal cancer susceptibility locus on 11q23 and replicates risk loci at 8q24 and 18q21	Albert Tenesa	Nature Genetics	80	2008
6	A genome-wide association study identifies colorectal cancer susceptibility loci on chromosomes 10p14 and 8q23.3	Ian Tomlinson	Nature Genetics	71	2008
7	A genome-wide association study shows that common alleles of SMAD7 influence colorectal cancer risk	Peter Broderick	Nature Genetics	66	2007
8	A genome-wide association scan of tag SNPs identifies a susceptibility variant for colorectal cancer at 8q24.21	Ian Tomlinson	Nature Genetics	64	2007
9	Genome-wide association scan identifies a colorectal cancer susceptibility locus on chromosome 8q24	Brent W. Zanke	Nature Genetics	63	2007
10	Meta-analysis of three genome-wide association studies identifies susceptibility loci for colorectal cancer at 1q41, 3q26.2, 12q13.13 and 20q13.33	Richard S. Houlston	Nature Genetics	62	2010

## Discussion

Bibliometrics is an interesting tool to access scientific outcomes of a specific academic field for a given period of time. After bibliometric analysis, a rich vein of useful reference information will be provided by its quantitative indexes. Simultaneously, it will predict some potential tendencies of one discipline for the future to guide more creative researchers. However, certain shortcomings do exist in bibliometric analysis. For instance, this analysis method based on the amounts of publications will inevitably ignore the quantities of papers of different levels. Obviously, bibliometrics cannot exclude those defunct scientific journals.

Within the 10 years covered by this study, the number of documents on SMAD7 in oncology has increased gradually, reaching a peak in 2020. There is still room for development and deepening for years to come. The distribution of publications by year shows that an increasing number of researchers are becoming enthusiastic about SMAD7 research. On the other hand, as numerous unsolved questions exist in the field of oncology, such as detailed mechanisms of key molecules, specific roles of related signal pathways, and the relationship between effective inferences and clinical manifestations, related studies might continue to be focal points in the future. Besides, along with the rapid development of social economy, more attention is focused on health care. According to Global Cancer Statistics 2020 published by International Agency for Research on Cancer (IARC) and Global Health Estimates 2020 from the World Health Organization (WHO) (Sung et al., 2021), cancer is regarded as a dominating cause of death all over the world. Besides being hazardous to human’s physiological and psychological health, cancer’s rising is also the barrier to increasing life expectancy. Due to there are still numerous undefined inducements and mechanisms of cancer existing, it is plausible that researchers make efforts to produce abundant outcomes in recent years. Absolutely, fresh ideas and amazing research achievements will inspire other researchers in adjacent scientific fields to go deep into their own subjects. After being adjusted to be a tendency line, the growth pattern of acquired publications was inclined to fit a linear equation rather than an exponential trend according to the correlation coefficient of 0.9819 of its linear equation y = 85.412x-1719800. Price’s Law is a kind of predictive index value using normex forecasting for a specified time duration. As time goes by, not every distribution of scientific papers complies with the law in any period of time. Even more, scientific publications’, as a subsystem of information dissemination, growth patterns are impacted and restricted by many aspects. The linear tendency of publications about SMAD7 in oncology indicates that the burst period of studies on SMAD7 has not yet come; there are still adequate spaces for further research on it.

However, the development of research across different countries or regions has been unbalanced, which may relate to the diversity of multilateral cooperation, susceptible populations, and the emphasis given to medical research. The most prolific organizations were in China, but the major institutions with the highest total citations were in the United States. It may be concluded that Chinese researchers, with the advantage of a large population, are equipped to conduct more research and publish more documents, while Western researchers are more influential in the study of oncology.

Based on the analysis results, prolific or influential researchers mostly belonged to organizations with high citations, and there was frequent cooperation and communication among influential authors. For example, Lan Huiyao, who came from The Chinese University of Hong Kong, ranked first with the highest number of citations on the list. Lan’s laboratory had a partnership with the No. 2 highest-citation researcher—Meng Xiaoming—at Anhui Medical University. Li Jun, also from Anhui Medical University, was also in cooperation with Meng. The authors mentioned above all had a cooperative relationship with Patrick M Tang from The Chinese University of Hong Kong. Therefore, there was a strong link between Anhui Medical University and The Chinese University of Hong Kong in the past decade. Additionally, among the top 10 authors with the highest number of citations, Hermann Brenner from Germany Cancer Research Center, collaborated with Andrew T Chan at Harvard Medical School. In terms of content, Lan’s research group has concentrated on cancer immunity in recent years (Li C. et al., 2020; Xue et al., 2020), the emphasis of Meng and Li’s team was on noncoding RNA (Bu et al., 2020; Xu et al., 2020), and Brenner’s laboratory has focused at the genome level (Corradi et al., 2021; Huyghe et al., 2021). Apparently, international collaboration is beneficial to producing abundant and influential papers. However, most Chinese investigators cooperated with other Chinese researchers, and there were few Chinese authors connected with Western laboratory teams. However, institutions from Germany, the United States, and the United Kingdom communicated closely with each other. Our bibliometric burst analysis results indicate that there many countries and regions that have continued to research SMAD7 in recent years. Moreover, a wide array of less-connected research groups is emerging outside this tight relation network, indicating that the role of SMAD7 in oncology is engaging increasing attention in this academic field.

In accordance with the keyword analysis, TGF-beta, NF-kappa-B, and beta-catenin were the most common molecules in studies about SMAD7 in oncology. Analysis of word frequency suggested that these core molecules are mostly involved in proliferation, epithelial-mesenchymal transition, or metastasis in breast cancer, colorectal cancer, or hepatocellular carcinoma. This indicates that these molecules or cancers are most commonly studied as research priorities, and they may be regarded as a complex topic for in-depth research. In addition to being the highest frequency word, TGF-beta was directly mentioned in the titles of 8 of the top 10 articles with the highest citation numbers. TGF-beta was also one of the top 20 keywords with the strongest citation burst from 2011 to 2013. If a longer timespan had been set in the search strategy, the beginning burst year of TGF-beta would probably be earlier than 2011. The TGF-beta signaling pathway plays a crucial role in the homeostasis and development of many species by regulating cell proliferation, migration, and differentiation; however, this signaling pathway suppresses the antitumor ability of immune cells in the tumor microenvironment (Huynh et al., 2019). It makes sense that TGF-beta was the highest frequency molecule on the list.

Moreover, far more genome studies than other subjects were highly ranked in terms of publication and citation numbers. Similarly, “genome-wide association” had the strongest burst strength from 2012 to 2015, followed by “susceptibility loci,” “genetic polymorphism,” and “single-nucleotide polymorphism.” Brenner’s team also published several genome-scale studies in 2021. This phenomenon indicates that investigators have paid more attention to intensive studies at the genetic level. Identification of specific risk loci is helpful to determine the etiologic heterogeneity of various cancers and to understand the risk factors and mechanisms of carcinogenesis.

Based on the results of keyword extraction and analysis, the dominant molecules or diseases mentioned in the top-ranked items may have been popular research topics over the past years but may slow down with the development of other novel directions. Meanwhile, these core words ranked lower or bursting in the most recent years may have the potential for explosive development in the future. PPAR-gamma, the abbreviation peroxisome proliferator activated receptor-gamma, is clearly a fresh keyword with a high citation burst emerging from 2018 and tends to be a continuous focus in the years ahead. In early studies, PPAR-gamma was known to be important for adipocyte differentiation, maintenance, and function (Lehrke and Lazar, 2005). Recent studies have shown that PPAR-gamma is involved in the proliferation of cells in various organs, such as the colon, breast, and bladder; moreover (dys)regulation of PPAR-gamma signaling pathways may participate in tumor occurrence and development in these organs (Lehrke and Lazar, 2005; Yousefnia et al., 2018). Additionally, as a crucial ligand-inducible transcription factor, PPAR-gamma may perform a variety of functions in the mechanisms of some therapeutic drugs (Cheng et al., 2019; Mansour et al., 2021). In early research, it was clear that PPAR-gamma is the dominant regulator of adipogenesis and can modulate metabolism and inflammation in immune cells (Tontonoz and Spiegelman, 2008). An increasing body of evidence suggests that some functional relationships do exist between cancer and inflammation. Although PPAR-gamma is expressed at a high level in adipose cells, substantial levels are also present in other cell types, such as colon, breast, and prostate cells. PPAR-gamma activation is related to a reduction in beta-catenin levels. Based on the well-established role of beta-catenin in carcinogenesis of the colon and other tissues, it is worth noting that PPAR-gamma generally works in opposition to the canonical WNT/beta-catenin signaling pathway (Vallee and Lecarpentier, 2018).

To some extent, the burst of PPAR-gamma studies emphasizes the significance of researching the correlations among inflammation, immunity, and cancer. High levels of proinflammatory factors contribute to angiogenesis, which is beneficial for carcinogenesis. Additionally, proinflammatory factors not only can suppress anticancer activities but also can help to create an environment that is conducive to DNA damage, consequently accumulating and amplifying carcinogenic effects. Cytokines produced by tumors can attract leukocytes together; in turn, leukocytes generate a series of cytokines, such as interleukin, tumor necrosis factor-alpha, interferon, etc. One of the most persuasive examples of the “relationship between inflammation and cancer” is that individuals with inflammatory bowel disease often later develop colorectal cancer (Herszenyi et al., 2015). The above-mentioned research achievements and trends explain why an increasing number of researchers dedicate themselves to shedding new light on PPAR-gamma and its role in the mechanisms of carcinogenesis.

From the summary of the current perspectives above, some potential prospects of SMAD7 in oncology have been gradually revealed to researchers. TGF-beta, one of the high-occurrence words, as well as the first of the top three focused-on molecules, is undoubtedly the primary research focus. Members of the TGF-beta signaling pathway are implicated in cellular functions including proliferation, apoptosis, cell dormancy, and autophagy, as well as cellular senescence (Zhang et al., 2017). TGF-beta signaling is also involved in several human cancers, such as breast cancer, colorectal cancer, and hepatocellular carcinoma (Fabregat et al., 2016; Itatani et al., 2019; Zhao et al., 2018). In particular, TGF-beta is likely to act as a double-edged sword in different phases of cancers, with antitumor effects in the early stage and protumor effects in the late stage (Roberts and Wakefield, 2003). The strong citation burst observed for TGF-beta similarly proves that this signaling pathway will continue to be a hotspot in the future.

In addition, novel mechanisms of miRNAs, circRNAs, lncRNAs, and posttranslational modifications have been shown to play key roles in carcinogenesis (Bhan et al., 2017; Chen et al., 2020; Li R. et al., 2020; Mishra et al., 2016). We can see that 5 of the top 20 keywords with the strongest citation bursts are associated with genomic research (genome-wide association, susceptibility loci, genetic polymorphism, single-nucleotide polymorphism, and microRNA expression). This citation burst list is further persuasive evidence that the research on SMAD7 has tended to focus on a genomic level.

Obviously, PPAR-gamma is the most notable burst term in recent years, as shown in Figure 7, with a sustainable development trend. A functional relationship does exist between cancer and inflammation (Coussens and Werb, 2002). PPAR-gamma exerts its anti-inflammatory effect by inducing proinflammatory factors, such as TGF-beta, TNF-alpha, NK-kappa-B, and IFN-gamma (Tontonoz and Spiegelman, 2008; Vallee and Lecarpentier, 2018). These key molecules are also high-frequency words in Table 5, further validating this potential research trend. Additionally, most of the key terms in Table 5 relate to inflammation, immunity, or cancer. Therefore, there is no doubt that research on the relationships among inflammation, immunity, and cancer will continue to be the main potential research hotspot in the future.

Although this is the first bibliometric study on SMAD7 in oncology, several potential limitations do exist in our study. The most obvious deficiency is that only documents mentioning SMAD7 in their titles, abstracts, or keywords were retrieved from the database, so there must be some relevant documents omitted, resulting in an incomplete analysis. Another limitation is that we chose the Web of Science Core Collection as the exclusive database to search due to its suitability for our visual analysis software. Although the Web of Science Core Collection is one of the most acknowledged databases among academics for searching important and influential publications, some publications are not included in this database and were inevitably missed. Then, due to the passage of time and the evolution of scientific research, bibliometric studies can provide a reference for only a relatively short period because the total citations, number of publications, and emerging keywords are changing frequently. Therefore, this study shows a complex structure at one point in time. Constant attention to fields of interest are necessary to keep pace with research trends. These shortcomings have also been mentioned in other bibliometric studies.

## Conclusion

This study is the first bibliometric paper focused on publications related to SMAD7 in the field of oncology worldwide. Through visual or cluster analysis, detailed information for numerous publications can be perceived more intuitively. There has been growth in research on SMAD7 over the past 10 years, especially from 2016 to 2019, and the number of documents will continue to increase in the future. The leading countries included China, the United States, and Germany. The organizations with the largest number of documents were Nanjing Medical University, Sun Yat-Sen University, and Shanghai Jiao Tong University. The most influential investigators were Lan Huiyao, Meng Xiaoming, and Hermann Brenner. The publication with the highest number of total citations was “Molecular mechanisms of epithelial-mesenchymal transition,” published by Samy Lamouille in 2014. More importantly, a prospective focus might be the specific mechanism of the interaction of PPAR-gamma with SMAD7 in oncology. Overall, this study summarizes data on published research papers and provides a visual reference for further studies in the field of oncology.

## Data Availability

The original contributions presented in the study are included in the article/Supplementary Material. Further inquiries can be directed to the corresponding author.

## Abbreviations

A&HCI, Arts and Humanities Citation Index; BMP, bone morphogenetic protein; Co-SMADs, common partner SMADs; CRC, colorectal cancer; IF, impact factors; IFN, interferon; I-SMADs, inhibitory SMADs; MeSH, medical subject headings; NF, nuclear factor; PPAR, peroxisome proliferator activated receptor; R-SMADs, receptor regulated SMADs; SCIE, Science Citation Index Expanded; SMAD, small mother against decapentaplegic; SSCI, Social Sciences Citation Index; TGF, transforming growth factor; TNF, tumor necrosis factor.
